# Intragenerational social mobility and self-rated oral health in the british cohort study

**DOI:** 10.1186/s12955-021-01757-1

**Published:** 2021-04-07

**Authors:** Aina Najwa Mohd Khairuddin, Eduardo Bernabé, Elsa Karina Delgado-Angulo

**Affiliations:** 1 Dental Public Health group, Faculty of Dentistry, Oral and Craniofacial Sciences, King’s College London, London, United Kingdom Bessemer Road, London, SE5 9RS UK; 2 Department of Health, Oral Health Division, Melaka, Malaysia; 3 Facultad de Estomatología, Universidad Peruana Cayetano Heredia, Lima, Peru

**Keywords:** Social mobility, Social class, Cohort studies, Adult, Oral health

## Abstract

**Background:**

Most studies on social mobility and oral health have focused on movement between generations (intergenerational mobility) rather than movement within an individual’s own lifetime (intragenerational mobility). The aim of this study was to investigate the association between intragenerational social mobility from early to middle adulthood and self-rated oral health.

**Methods:**

This study used data from 6524 participants of the 1970 British Birth Cohort Study, an ongoing population-based birth cohort of individuals born in England, Scotland and Wales. Participants’ socioeconomic position was indicated by occupational social class at age 26 and 46 years (the first and latest adult waves, respectively). Self-rated oral health was measured at age 46 years. The association between social mobility and adult oral health was assessed using conventional regression models and diagonal reference models, adjusting for gender, ethnicity, country of residence and residence area.

**Results:**

Over a fifth of participants (22.2%) reported poor self-rated oral health at age 46 years. In conventional regression analysis, the odds ratios for social mobility varied depending on whether they were adjusted for social class of origin or destination. In addition, all social trajectories had greater odds of reporting poor oral health than non-mobile adults in class I/II. In diagonal reference models, both upward (Odds Ratio 0.79; 95% CI 0.63–0.99) and downward mobility (0.90; 95% CI 0.71–1.13) were inversely associated with poor self-rated oral health. The origin weight was 0.48 (95% CI 0.33–0.63), suggesting that social class of origin was as important as social class of destination.

**Conclusion:**

This longitudinal analysis showed that intragenerational social mobility from young to middle adulthood was associated with self-rated oral health, independent of previous and current social class.

## Background

In social mobility research, a distinction is traditionally made between movement between generations (intergenerational) and within an individual’s own lifetime (intragenerational) [1]. Both forms of mobility are equally important for research and policy to address inequalities [2, 3]. While intergenerational mobility tells about how parents’ socioeconomic circumstances influence their children’s life (usually through educational attainment and school-to-work transition), intragenerational mobility tells about whether individuals can move into a different economic situation during adulthood (within-career mobility) [4, 5]. The extent of intragenerational mobility within the labour market is a measure of opportunities and consistency in careers because it affects the development of human capital and wage trajectories [5].

Several theories have been proposed to explain mobility effects on health, such as the *dissociative theory* which posits that any mobility (upward or downward) is a strenuous experience for mobile individuals as they face a conflict between previous and new social ties [6]; the *rising from rags argument* which states that upward mobility may be conducive to greater well-being due to fulfilled aspirations and improved feelings of self-esteem and self-control [7]; the *falling from grace thesis* which proposes that downward mobility is harmful to health due to feelings of failure and insecurity [8]; the *status maximization hypothesis* which postulates that individuals adjust to the highest available status group (either past or current) [9]; and the *acculturation theory* which claims that health of mobile individuals is a result of being in different class contexts rather than mobility itself, with destination playing a bigger role than origin [10].

The effect of social mobility on adult oral health has been investigated in several longitudinal studies, although most have focused on intergenerational mobility [11–20]. Only two previous studies have looked at intragenerational mobility in relation to oral health [12, 21]. MS Pearce, WM Thomson, AW Walls and JG Steele [12] found that adults who were persistently low and downwardly mobile between ages 25 and 50 years were less likely to retain a functional dentition than the persistently high group, although no differences in oral health-related quality of life were found between the four trajectory groups [12]. SE Ramsay, E Papachristou, RG Watt, LT Lennon, AO Papacosta, PH Whincup and SG Wannamethee [21] showed that participants who were persistently low and upwardly mobile between middle adulthood (40–59 years) and older age (71–92 years) were more likely to have fewer teeth and poor self-rated oral health, but not periodontal disease, than those in the persistently high group, after accounting for childhood socioeconomic conditions. No formal assessment of theories of mobility effects on oral health have been conducted to date. Such an assessment is important, from a sociological viewpoint, because it shed lights on the specific mechanisms through which social mobility affects people’s oral health. Indeed, there is evidence that social mobility effects manifest differently depending on the health outcome [2, 22].

The traditional analytical approach to study social mobility effects has been to classify individuals based on their social origin and destination groups (for instance as persistently high, upwardly mobile, downwardly mobile and persistently low derived from binary indicators at baseline and follow-up) and compare their health outcomes. However, this approach does not disentangle the mobility effect from those of past and current social groups [23, 24]. An alternative is the use of diagonal reference models, which assume those who remain stable over time are the benchmark group and the health outcome of socially mobile individuals lies somewhere within the social group of origin and the social group of destination [24, 25].

Using diagonal reference models, this study investigated the association between intra-generational social mobility from early to middle adulthood and self-rated oral health. The study also assessed which of the five theories of mobility effects on health (dissociative theory, rising from rags, falling from grace, status maximization and acculturation) were better supported by the data. Subjective health assessments are a legitimate summary of individuals’ clinical and self-reported health, representing a valid, reliable and cost-effective health assessment method for epidemiological surveys [26, 27].

## Methods

### Data source

This study used data from the 1970 British Cohort Study, an ongoing population-based birth cohort of around 17,000 people, who were born in England, Scotland and Wales within a single week of April 1970. There have been 10 waves of data collection to date, with information collected from parents at birth and during childhood whereas the cohort members started to provide information from later childhood [28]. For this study, data from waves 5 and 10 were used (ages 26 and 46 years). Wave 5 was the first adult follow-up of participants (carried out in 1996), and represents the age by which most individuals had completed their full-time education and are expected to enter the labour market [29]. Wave 10 was the latest follow-up and contained oral health data (carried out in 2016). The retention rate was 76% at age 26 years and 70% at age 46 years. An evaluation of attrition showed that disadvantaged groups, who are at higher risk of poor health too, are underrepresented [30].

**Fig. 1:**
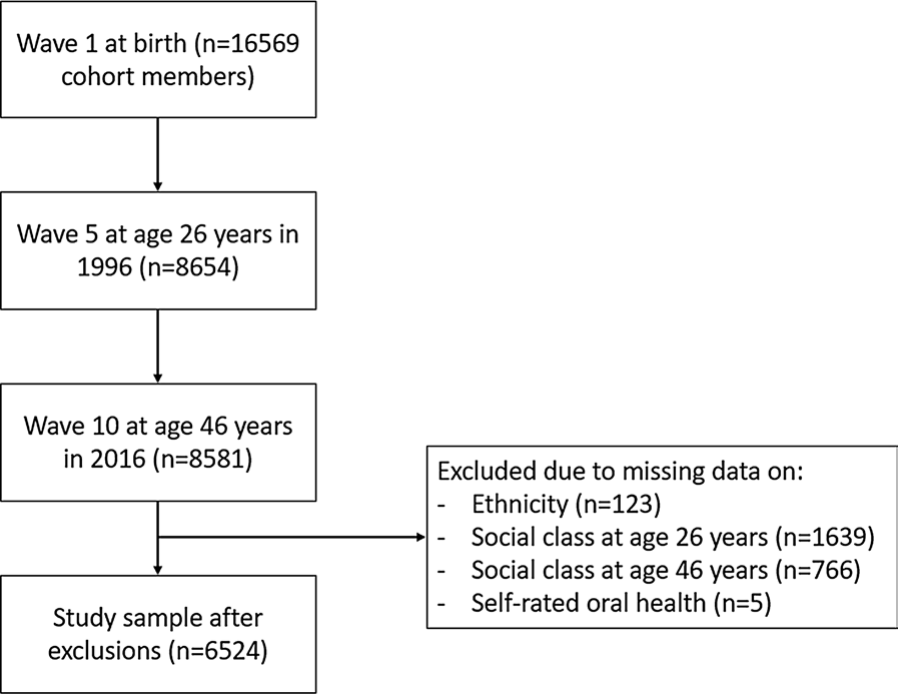
Flowchart of the 1970 British Cohort Study and derivation of the study sample

Of the 8581 participants with a productive questionnaire at age 46 years, 2143 were excluded for missing data on oral health (n = 5), social class at age 26 years (n = 1639), social class at age 46 years (n = 766) and confounders (ethnicity = 123). Therefore, the study sample included 6524 individuals (Fig. 1).

### Measurements

Self-rated oral health was the outcome, which was measured with the question “Would you say that your dental health (your mouth, teeth and/or denture) is excellent, very good, good, fair or poor?” [31]. It was the only question on oral health included in the wave 10 questionnaire. This global oral health item has been shown to correlate well with clinical measures of oral diseases [27, 32] and it has been used in national surveys [33]. Responses were grouped into two categories: good for those who responded excellent, very good or good (reference group) and poor for those who responded fair or poor.

Intragenerational social mobility was the exposure of interest, derived from participants’ social class at ages 26 (class of origin) and 46 years (class of destination). At each age, occupational social class was determined according to the Registrar General’s Social Class, which groups occupations in six hierarchical classes based on their prestige: professional (I), managerial and technical (II), skilled non-manual (III-NM), skilled manual (III-M), partly skilled (IV) and unskilled (V) [34]. Participants who were unemployed in one or both waves were excluded from the analysis. The six categories were merged into three: class I/II (highest), class III-NM/M (middle) and class IV/V (lowest) [35]. From these two measures, three indicators were created to capture the movement between ages 26 and 46 years. The first indicator reflected any mobility (coded as 1 for mobile participants and 0 for non-mobile participants), the second indicator reflected upward mobility (coded as 1 for participants who moved from a lower to a higher class over time and 0 otherwise) and the third indicator reflected downward mobility (coded as 1 for those who moved from a higher to a lower class over time and 0 otherwise).

Variables associated with both intragenerational mobility and oral health, based on previous relevant studies [2, 3, 12, 21], were chosen to control for confounding. They were participants’ gender, ethnicity (White versus non-White), country of residence (England, Wales and Scotland) and residence area (urban versus rural). Participants’ education was not included as it overlaps closely with social class.

### Statistical analysis

All analyses were run in Stata 16.0 (StataCorp, College Station, TX). First, participants in the study sample and those excluded because of missing data were compared in terms of self-rated oral health, social class at ages 26 and 46 years and confounders (gender, ethnicity, country of residence and residence area). Thereafter, the proportion of participants reporting poor oral health was estimated for each possible cell in a contingency table of social class at age 26 and 46 years.

The association between intragenerational social mobility and self-rated oral health was assessed using standard regression and diagonal reference models. For comparison purposes, standard specifications previously reported in the literature were first fitted. In the first specification (labelled as Model 1), an indicator for the class of origin (or the class of destination) was entered simultaneously with an indicator for social mobility (stable, upward and downward) in the regression model [36, 37]. In the second specification (Model 2), a variable including every possible trajectories from origin to destination (I/II to I/II, IIINM/M to IIINM/M, IV/V to IV/V, I/II to IIINM-M, I/II to IV/V, IIINM/M to IV/V, IIINM/M to I/II, IV/V to I/II and IV/V to IIINM/M) was used to represent social mobility [12, 21]. Diagonal reference models were used to separate the effects of the social groups of origin and destination from the effect of mobility itself [23, 24], which cannot be done with conventional regression models because they are linearly dependent (social mobility = destination group – origin group) [25]. Diagonal reference models estimate the probability of having poor oral health *Y*_*ijk*_ of individual *k* in cell *ij* in a contingency table of class of origin *i* by class of destination *j* as the weighted sum of the estimated probability of poor oral health in the non-mobile groups at origin (μ_ii_) and destination (μ_jj_). These weights are the non-linear product terms *q* and *(1-q)*, which represent the relative influence of the classes of origin and destination, respectively. They are constrained to be positive and sum to one. The diagonal effects μ_11_, μ_22_ and μ_33_ in the contingency table correspond to the social gradient in poor oral health for non-mobile individuals [23–25]. Four alternative specifications were tested: Model 3 only included the effects of class of origin and destination; Model 4 included an indicator for any mobility (upward or downward); Model 5 included an indicator for upward mobility; and Model 6 included an indicator for downward mobility. The fit of the models was evaluated using the Akaike and Bayesian Information Criteria [25]. Results from these models supported (i) the dissociative theory if any mobility was positively associated with poor oral health (Model 4), (ii) the rising from rags argument if upward mobility was inversely associated with poor oral health (Model 5); (iii) the falling from grace thesis if downward mobility was positively associated with poor oral health (Model 6); (iv) the status maximization hypothesis if upward mobility was inversely associated with poor oral health and downward mobility was inversely or not associated with poor oral health (Models 5 and 6, respectively); and the acculturation theory if social mobility was not associated with poor oral health in Models 4, 5 and 6 and the destination weight was higher than the origin weight in Model 3 [2, 3, 7]. All regression models were fitted using binary logistic regression and adjusted for the same set of confounders (gender, ethnicity, country of residence and residence area). Odds ratios (ORs) with 95% confidence intervals (CI) were reported as the measure of association.

## Results

Data from 6524 participants were analysed. Participants excluded from the analysis were more likely to be female and non-White, live in Scotland and urban areas, have low social class at ages 26 and 46 years and report poor oral health (Table 1). Slightly over a fifth of participants (22.2%) reported poor oral health.

**Table 1:** Characteristics of participants in the study sample (n = 6524) and comparison with those excluded because of missing values (n = 2057)

		Study sample		Excluded	
		n	%	n	%
*Gender*					
Male		3302	50.6	852	41.4
Female		3222	49.4	1205	58.6
*Ethnicity*					
White		6232	95.5	1797	92.9
Non-White		292	4.5	137	7.1
*Country*					
England		5642	86.5	1805	87.7
Wales		546	8.4	122	5.9
Scotland		336	5.2	130	6.3
*Residence area*					
Urban		4506	69.1	1534	74.6
Rural		2018	30.9	523	25.4
*Social class at age 26 years*					
I/II		2724	41.8	148	35.4
III-NM/M		2882	44.2	178	42.6
IV/V		918	14.1	92	22.0
*Social class at age 46 years*					
I/II		3586	55.0	545	42.2
III-NM/M		1797	27.5	390	30.2
IV/V		1141	17.5	356	27.6
*Self-rated oral health*					
Good		5075	77.8	1358	66.2
Poor		1449	22.2	694	33.8

Table 2 shows the proportion reporting poor oral health in nine groups of individuals, defined by the joint distribution of the social class of origin and social class of destination. The main diagonal shows a clear social gradient in poor oral health among non-mobile adults. In addition, poor oral health was more common among downwardly mobile than among upwardly mobile individuals. Clear gradients in poor oral health by social class can also be observed in every row and column of the contingency table.

**Table 2:** Prevalence of poor self-rated oral health at age 46 years by social class or origin (age 26) and destination (age 46 years) in the 1970 British Cohort Study (n = 6524)

Social class at age 26 years		Social class at age 46 years			
		I/II	III-NM/M	IV/V	Total
*Prevalence of poor oral health*					
I/II		17.8	19.0	19.8	18.4
III-NM/M		18.1	25.3	30.4	23.4
IV/V		25.0	30.7	34.6	30.6
Total		18.5	24.7	30.1	22.2
*Cell sizes (n)*					
I/II		2136	401	187	2724
III-NM/M		1170	1139	573	2882
IV/V		280	257	381	918
Total		3586	1797	1141	6524

Table 3 presents the results from conventional regression analysis. The odds ratios for social mobility varied depending on whether it was entered simultaneously with social class at age 26 (Model 1A) or 46 years (Model 1B). Upwardly mobile adults had 34% (OR: 0.66; 95%CI: 0.57–0.78) lower odds while downwardly mobile adults had 1.24 (95%CI: 1.05–1.45) times greater odds of reporting poor oral health when adjusted for social class of origin. However, upwardly mobile adults had 1.18 (95%CI: 1.02–1.37) times greater odds while downwardly mobile adults had 29% (0.71; 95%CI: 0.59–0.86) lower odds of reporting poor oral health when adjusted for social class of destination. When using the indicator with nine possible trajectories from age 26 to age 46 years (Model 2), all trajectory groups had greater odds of reporting poor oral health than non-mobile adults in class I/II. The largest odds ratios were found among non-mobile adults in class IV/V (2.52; 95%CI: 1.98–3.20), downwardly mobile adults from class III-NM/M to IV/V (2.06; 95%CI: 1.67–2.55) and upwardly mobile adults from class IV/V to III-NM/M (2.05; 95%CI: 1.54–2.74).

**Table 3:** Conventional specifications for testing the association between social mobility and poor self-rated oral health using logistic regression (n = 6524)

		Model 1A OR^a^ [95% CI]	Model 1B OR^a^ [95% CI]	Model 2 OR^a^ [95% CI]
*Social class at age 26 years*				
I/II		1.00 [Reference]		
III-NM/M		1.64 [1.42, 1.88]		
IV/V		2.65 [2.19, 3.23]		
*Social class at age 46 years*				
I/II			1.00 [Reference]	
III-NM/M			1.64 [1.42, 1.90]	
IV/V			2.60 [2.12, 3.18]	
*Social mobility*				
Stable		1.00 [Reference]	1.00 [Reference]	
Upwardly mobile		0.66 [0.57, 0.78]	1.18 [1.02, 1.37]	
Downward mobile		1.24 [1.05, 1.45]	0.71 [0.59, 0.86]	
*Social trajectories*				
I/II to I/II				1.00 [Reference]
III-NM/M to III-NM/M				1.61 [1.35, 1.91]
IV/V to IV/V				2.52 [1.98, 3.20]
I/II to III-NM/M				1.13 [0.86, 1.48]
I/II to IV/V				1.25 [0.86, 1.83]
III-NM/M to IV/V				2.06 [1.67, 2.55]
III-NM/M to I/II				1.05 [0.87, 1.27]
IV/V to I/II				1.59 [1.18, 2.13]
IV/V to III-NM/M				2.05 [1.54, 2.74]

^a^Logistic regression was fitted and odds ratios (OR) were adjusted for gender, ethnicity, country of residence and residence area. Model 1A included separate indicators for social class at age 26 years and social mobility from age 26 to 46 years. Model 1B included separate indicators for social class at 46 years and social mobility from age 26 to 46 years. Model 2 included nine possible social trajectories from age 26 to age 46 years

Table 4 reports the estimates from diagonal reference models. Model 3, which included social class at origin and destination but no indicator for social mobility, confirms the social gradient observed in Table 2, even after adjustments. The odds of reporting poor oral health among non-mobile individuals were 0.61 at class I/II, 0.99 at class III-NM/M and 1.65 at class IV/V. The weights for origin at 0.48 (95%CI: 0.33–0.63) and destination at 0.52 (95%CI: 0.37–0.67) indicate that past social class is as important as current social class. Model 4 shows that mobility in any direction was associated with 10% (0.90; 95%CI: 0.80–1.02) lower odds of reporting poor oral health. Models 5 and 6 distinguish between upward and downward mobility, respectively. Upwardly mobile adults had 21% lower odds (0.79; 95%CI: 0.63–0.99) of reporting poor oral health than non-mobile individuals (Model 5) whereas downwardly mobile individuals had 10% (0.90; 95%CI: 0.71–1.13) lower odds of reporting poor oral health than non-mobile individuals (Model 6), even after adjustment for class of origin, class of destination and confounders. Model 5 (upward mobility) had the best fit according to the Akaike and Bayesian Information Criteria. In stratified analysis by sex, findings from the diagonal reference models were consistent for men and women.

**Table 4:** Diagonal reference models for the association between social mobility and poor self-rated oral health among participants in the 1970 British Cohort Study (n = 6524)

		Model 4 OR^a^ [95% CI]	Model 5 OR^a^ [95% CI]	Model 6 OR^a^ [95% CI]	Model 7 OR^a^ [95% CI]
*Weights*^*b*^					
p: Origin		0.48 [0.33–0.63]	0.50 [0.35–0.64]	0.67 [0.44–0.91]	0.41 [0.19–0.62]
1-p: Destination		0.52 [0.37–0.67]	0.50 [0.36–0.65]	0.33 [0.09–0.56]	0.59 [0.38–0.81]
*Diagonal intercepts (non-mobile individuals)*^*c*^					
μ_11_: I/II		0.61 [0.55–0.68]	0.60 [0.54–0.66]	0.60 [0.54–0.66]	0.61 [0.55–0.67]
μ_22_: III-NM/M		0.99 [0.89–1.11]	0.99 [0.89–1.11]	0.99 [0.90–1.11]	0.99 [0.89–1.12]
μ_33_: IV/V		1.65 [1.45–1.88]	1.68 [1.48–1.92]	1.67 [1.48–1.90]	1.67 [1.46–1.90]
*Control variables*					
*Gender (reference: male)*					
Female		0.58 [0.52–0.66]	0.59 [0.52–0.66]	0.59 [0.52–0.66]	0.59 [0.52–0.66]
*Ethnicity (reference: white)*					
Non-White		1.06 [0.80–1.42]	1.06 [0.80–1.42]	1.06 [0.80–1.42]	1.06 [0.80–1.42]
*Country (reference: England)*					
Scotland		1.08 [0.87–1.35]	1.08 [0.87–1.35]	1.08 [0.87–1.35]	1.08 [0.87–1.35]
Wales		1.08 [0.84–1.42]	1.08 [0.83–1.42]	1.08 [0.83–1.40]	1.08 [0.84–1.42]
*Residence area (reference: urban)*					
Rural		0.86 [0.76–0.99]	0.87 [0.76–0.99]	0.87 [0.76–0.99]	0.86 [0.76–0.99]
*Any mobility (reference: stable)*					
Mobile			0.90 [0.80–1.02]		
*Upward mobility (reference: no)*					
Yes				0.79 [0.63–0.99]	
*Downward mobility (reference: no)*					
Yes					0.90 [0.71–1.13]
*Model fit statistics*					
AIC		6745.1	6744.5	6743.0	6746.2
BIC		6806.1	6812.3	6810.9	6814.0

AIC: Akaike Information Criterion; BIC: Bayesian Information Criterion; CI: Confidence interval

^a^Diagonal reference models were fitted with a logit link and odds ratios (ORs) were thus reported for all control variables. Model 4 did not include any indicator for social mobility (baseline for comparison); Model 5 included an indicator for any mobility (upward or downward); Model 5 included an indicator for upward mobility; and Model 6 included an indicator for downward mobility

^b^Weights for social class of origin and destination are expressed as coefficients from 0 to 1

^c^These values are odds of reporting poor oral health among non-mobile adults at each social class

## Discussion

This study shows that social mobility from young to middle adulthood was associated with self-rated oral health among British individuals, after accounting for social class of origin, social class of destination and demographic characteristics. Interestingly though, both upward and downward mobility were positively associated with lower odds of reporting poor oral health. The findings also show that the social classes of origin and destination had an equal influence on participants’ oral health.

The findings from diagonal reference models are in clear contrast with those from conventional regression models, which showed that the association between social mobility and self-rated oral health varied depending on whether adjustment was made for social class of origin or destination (Model 1A-1B in Table 3) or that all upward and downward trajectories were associated with poorer oral health (Model 2 in Table 3). These differences using the same data stem from the intrinsic limitations of the conventional regression models, which conflate the effects of origin, destination and mobility during estimation, thus yielding biased results [25]. These limitations can also explain the different conclusions reached by previous studies looking at intra-generational mobility and adult oral health [12, 21].

Our findings lend support to the *status maximization hypothesis* which claims that upwardly mobile individuals tend to adapt their values and lifestyle to their destination class whereas downwardly mobile individuals tend to be more influenced by their origin class [9, 38]. In line with this hypothesis we found that both upward and downward mobility were associated with lower odds of reporting poor oral health. We can thus speculate that upwardly mobile individuals would reflect the healthier lifestyle of their achieved social class (including health-promoting behaviours, better access and use of dental services and safer environments) whereas downwardly mobile individuals would be aware of the negative health effects of the lifestyle of their new social class and retain the lifestyle of their social class of origin. Our findings on upward mobility also give support to the *rising from rags argument* [7] insofar as they help explain the better oral health reported by upwardly mobile individuals. The two theories are not in opposition and it is possible that both play a role in explaining the beneficial effect of upward mobility on self-rated oral health. It must be kept in mind though that we used data from a young and healthy cohort of participants. As most chronic conditions start developing from older adulthood and occupational stability is achieved during that same period of life [29], it is possible that the effect of social mobility on oral health may vary when extending the assessment beyond middle adulthood. This has implications for the relevance of other theories as participants get older.

The findings have some implications for policy and research. They suggest that any form of social mobility (either upward or downward) might positively impact adult perceptions of oral health, probably because mobile individuals seek to maximise their status [9, 38]. The positive effect of downward social mobility found in this study contrasts with previous dental studies that reported a negative association between downward mobility and good oral health [11–21]. The fact that downward mobility was inversely associated with poor oral health implies that there will be more people with good oral health in the lower social class. Both processes, not only upward mobility, can therefore dilute social inequalities in oral health. As this is a young cohort, the findings also imply that socioeconomic conditions in early adulthood (from age 26 to 46 years as measured in this study) are relevant to perceived oral health. As for research, there is a need to revise findings from previous longitudinal studies on both inter- and intragenerational social mobility and oral health using diagonal reference models. This area would benefit from longitudinal studies using alternative socioeconomic indicators as well as other oral health outcomes (particularly clinical indicators) measured multiple times over the life course. More research is also required to further explore the plausible pathways through which social mobility affects oral health.

The strengths of this study were the use of prospectively collected data from a population-based cohort and the use of diagonal reference models to separate out the effects of social mobility from those of class of origin and destination. The study also had some limitations. First, about a quarter of participants were excluded from the analysis because of missing data on relevant variables. That said, most were excluded because they had no occupation at age 26 and/or 46 years. This is a well-known issue with occupational social class, which does not fully cover all the economically active population (such as those in long-term unemployment or who have never worked). As such, the present findings represent valid relationships between the variables of interest but are only applicable to participants who were in paid employment from young to middle adulthood. Second, we collapsed adjacent social classes due to small numbers in some cells, which created misclassification as mobility from class I to II, IIINM to IIIM, and IV to V (or vice versa) were not captured in our analysis (there were 7.8% upwardly mobile and 7.6% downwardly mobile participants in our non-mobile groups). However, similar findings were obtained when using four social classes, suggesting that such misclassification might have not affected the findings. Third, we did not have information on the age at which individuals moved up or down the social ladder. Social mobility effects could vary according to the amount of time spent in the class of origin and destination [2, 39]. Finally, we used a subjective indicator to characterise participants’ oral health that was measured at a single point in time. Our findings await confirmation from further studies using other oral health outcomes measured multiple times over the life span.

## Conclusion

Using diagonal reference models, this study found an association between intragenerational social mobility from young to middle adulthood and self-rated oral health among British adults that was independent of both prior and current social class. Our findings show that multiple theories on social mobility effects coexist in relation to oral health, and that more than one can be used to explain the findings.

## Data Availability

The datasets used in this study are publicly available from the UK Data Archive.

## Abbreviations

AIC: Akaike Information Criterion
BIC: Bayesian Information Criterion
CI: Confidence interval
NM/M: Non-manual/manual
ORs: Odds ratios
TX: Texas
